# Comparison of In Vitro and In Planta Heavy Metal Tolerance and Accumulation Potential of Different *Armeria maritima* Accessions from a Dry Coastal Meadow

**DOI:** 10.3390/plants11162104

**Published:** 2022-08-12

**Authors:** Līva Purmale, Astra Jēkabsone, Una Andersone-Ozola, Andis Karlsons, Anita Osvalde, Gederts Ievinsh

**Affiliations:** 1 Department of Plant Physiology, Faculty of Biology, University of Latvia, 1 Jelgavas Str., LV-1004 Rīga, Latvia; 2 Institute of Biology, University of Latvia, 4 Ojāra Vācieša Str., LV-1004 Rīga, Latvia

**Keywords:** *Armeria maritima*, heavy metals, metal accumulation potential, metal tolerance, phytoremediation, plant tissue culture

## Abstract

The aim of the present study was to compare the tolerance to several heavy metals and their accumulation potential of *Armeria maritima* subsp. *elongata* accessions from relatively dry sandy soil habitats in the Baltic Sea region using both in vitro cultivated shoot explants and long-term soil-cultivated plants at the flowering stage as model systems. The hypothesis that was tested was that all accessions will show a relatively high heavy metal tolerance and a reasonable metal accumulation potential, but possibly to varying degrees. Under the conditions of the tissue culture, the explants accumulated extremely high concentration of Cd and Cu, leading to growth inhibition and eventual necrosis, but the accumulation of Pb in their tissues was limited. When grown in soil, the plants from different accessions showed a very high heavy metal tolerance, as the total biomass was not negatively affected by any of the treatments. The accumulation potential for heavy metals in soil-grown plants was high, with several significant accession- and metal-related differences. In general, the heavy metal accumulation potential in roots and older leaves was similar, except for Mn, which accumulated more in older leaves. The absolute higher values of the heavy metal concentrations reached in the leaves of soil-grown *A. maritima* plants (500 mg Cd kg^−1^, 600 mg Cu kg^−1^, 12,000 mg Mn kg^−1^, 1500 mg Pb kg^−1^, and 15,000 mg Zn kg^−1^) exceeded the respective threshold values for hyperaccumulation. In conclusion, *A. maritima* can be characterized by a species-wide heavy metal tolerance and accumulation potential, but with a relatively high intraspecies diversity.

## 1. Introduction

The phenomenon of plant heavy metal tolerance has attracted the interest of plant ecologists and physiologists for several decades. From a practical point of view, the ability of tolerant plants to accumulate metals in their tissues, especially in their above-ground parts, is a characteristic that determines their potential use in environmental remediation technologies [1,2]. A relatively large number of such species has been identified and their phytoremediation potentials explored [3]. Still, comparative studies on the diversity of the functional mechanisms of metal tolerance and accumulation, as well as on the relative distribution of these plants in different ecosystems, are relatively scarce. It is becoming clear that plants with intriguing properties suitable as models in metal tolerance and accumulation studies can also be found in soil types other than metalliferous soils [4,5,6]. In fact, many metal-tolerant and highly accumulating genotypes have evolved as a result of local genetic adaptation in mine sites or in soils with a naturally high metal content. In addition, species-wide metal tolerance has been described, such as with *Typha latifolia* [7] and other species [4].

*Armeria maritima* (Mill.) Willd. is a perennial rosette-forming species with a complicated taxonomy—the frequent occurrence of hybridization being the main problem. Therefore, in Europe, *A. maritima* populations are characterized by continuous morphological variation, but several distinct groups have been initially defined: *A. maritima* subsp. *maritima*, *A. maritima* subsp. *elongata*, and *A. maritima* subsp. *alpina* [8]. At present, the later has been raised to the species rank [9]. However, populations from metalliferous soils (*A. maritima* subsp. *halleri* s.l.) show relatively different morphological traits, with each specific population being associated with a particular mine site. Initially, as based on morphological differences and ecogeographical distribution, *A. maritima* subsp. *maritima* was defined as an Atlantic taxon, occurring along coasts of north-west Europe, but *A. maritima* subsp. *elongata* as a continental taxon occurring around the Baltic Sea in dry sandy soils [10]. However, the former is considered to be a synonym of *A. maritima*, while the later retains its subspecies rank. Instead of getting involved in detailed taxonomic evaluation, other authors emphasize only ecological requirements of representatives of a particular population of *A. maritima* s.l., distinguishing ecotypes associated with salt marsh (also known as *A. maritima* subsp. *maritima*), dry sandy soils (also known as *A. maritima* subsp. *elongata*), and metalliferrous soils (also known as *A. maritima* subsp. *halleri*) [11].

*A. maritima* has been suggested to represent a “local metallophyte”, a species occurring both in metal-contaminated soils within a given region as well as in non-contaminated soils in distinct phytogeographic areas, in contrast to a “pseudometallophyte” species, being present in both contaminated and non-contaminated soils of the same region [12]. It is suggested that local populations of *A. maritima* in metalliferous soils have repeatedly evolved from nearby non-metalliferous soil populations [9], showing the significant species-wide basal potential for heavy metal tolerance. Moreover, epigenetic processes might be involved in development of metallicolous populations [13].

There is evidence indicating that in natural habitats, *A. maritima* plants have competitive ability only in relatively unfavorable conditions, well outside the optimum for the majority of the species. Species tolerance to water shortages [14], soil salinity [11], and a high metal concentration [15,16] seems to be the main characteristics in this respect. Thus, *A. maritima* has been documented as a predominant biomass-producing species in soils most polluted with Zn (5.7 to 4.1 g kg^−1^ of a plant-available metal), specifically a former zinc-smelting site, which disappeared when the metal concentration decreased (0.4 to 2.1 g kg^−1^) [17]. In addition, in a heavy metal-rich dry grassland complex near a Cu mine, the occurrence of *A. maritima* ssp. *halleri* positively correlated with the soil’s Cu concentration [18]. Moreover, in a metalliferous region, *A. maritima* did not occur in soils with ammonia acetate-soluble (plant-available) Pb less than 4 to 7 mmol (0.824 to 1.442 g), and it was suggested that this resulted from the low competitiveness of the species in low Pb soils [12].

Some studies have compared the heavy metal tolerances of *A. maritima* accessions from both metalliferous and non-metalliferous soils. However, the obtained results seem to be rather controversial. Short-term hydroponic experiments with *A. maritima* seedlings have shown that plants from a Zn-Pb polluted site had a higher tolerance to Zn, Cd, and Pb in comparison to plants from an unpolluted site, and the majority of these heavy metals accumulated in the roots of plants from both sites [19]. In contrast, during a long-term cultivation in artificial soil, *A. maritima* plants from both metalliferous and non-metalliferous sites showed similar Zn tolerances with no growth inhibition for up to 2.8 mmol kg^−1^ [15]. Still, plants native to metalliferous soils accumulated more Zn in comparison to these from non-metalliferous soils, predominantly in roots.

While tissue culture of *A. maritima* has been thoroughly investigated [20] and has been used as a means for the propagation of *A. maritima* plants for heavy metal remediation experiments [21], no study so far has explored and compared the heavy metal responses and accumulation potential of *A. maritima* in tissue cultures with those of whole plants. However, tissue culture experiments with heavy metals have been performed with a number of other model plants: *Brassica* spp. [22], *Populus* spp. [23,24], *Prunus cerasifera* [25], etc.

The aim of the present study was to compare the tolerance to several heavy metals and the accumulation potential of *A. maritima* subsp. *elongata* accessions from relatively dry sandy soil habitats in the Baltic Sea region. Both in vitro cultivated shoot explants and long-term soil-cultivated plants at the flowering stage were used as model systems. It was hypothesized that all the accessions would show a relatively high heavy metal tolerance and a reasonable metal accumulation potential, but possibly to varying degrees.

## 2. Materials and Methods

### 2.1. Plant Material

Seeds of *A. maritima* subsp. *elongata* from three geographically isolated micropopulations growing in sandy soils in water reservoir-associated meadows were used as propagation material (Table 1). Seeds of AM1 and AM2 were used for initiation of tissue culture as described further. Multiplied shoot explants were used for tissue culture experiment or were rooted and acclimatized for soil culture experiment. Seeds of AM3 were used for establishment of plants for soil culture experiment.

### 2.2. Tissue Culture

Seeds (30 from each accession) were surface-sterilized with a commercial bleach ACE (Procter & Gamble, Warszawa, Poland; diluted with sterile water 1:1) with a drop of Tween 40 for 10 min followed by three washes with sterile deionized water. Surface sterilized seeds were germinated in 200 mL glass jars on half-strength Murashige and Skoog (MS) [26] medium supplemented with sucrose (30 g L^−1^) and agar (6 g L^−1^). Prior autoclaving pH was adjusted to 5.8 [27]. Seeds were germinated in a growth cabinet under 16-h photoperiod provided by a fluorescent light with photon flux density 50 μmol m^−2^ s^−1^ of photosynthetically active radiation at 25 °C. After 45 days, seedlings were transferred to multiplication medium. For multiplication, full strength MS medium supplemented with 0.1 mg L^−1^ 1-naphthaleneacetic acid and 1 mg L^−1^ 6-benzylaminopurine, sucrose (30 g L^−1^), and agar (6 g L^−1^) was used. Every 4–5 weeks developed shoots were divided and transferred to fresh medium. For rooting, MS medium was supplemented with 0.2 mg L^−1^ 1-naphthaleneacetic acid, sucrose (30 g L^−1^), and agar (6 g L^−1^) [28]. Rooted plantlets were transferred to peat substrate and, after two weeks, were fully acclimatized ex vitro.

### 2.3. Experiment 1: Tissue Culture with AM1 and AM2

Salts of heavy metals Cd, Cu, Mn, Pb, and Zn were added to multiplication medium before autoclaving (Table 2). Medium was poured in 200 mL jars (five per treatment) and five shoot explants were placed in each jar. Cultures were placed in a growth cabinet under 16-h photoperiod provided by a fluorescent light with photon flux density 50 μmol m^−2^ s^−1^ of photosynthetically active radiation at 25 °C. After 4 weeks, the experiment was terminated, and number of necrotic explants was evaluated. Multiplication rate was evaluated according to the number of new shoots formed (0, no shoots; 1, 1–2 shoots; 2, 3–5 shoots; 3, >5 shoots). Fresh and dry mass (after drying at 60 °C for 6 days) of tissues were measured. Tissue water content was expressed as g of H_2_O per g dry mass.

### 2.4. Experiment 2: Soil Culture with AM1 and AM2

Acclimatized plants propagated in tissue culture were individually planted in 1.3 L plastic containers filled with 1 L of a mixture of quartz sand (Saulkalne S, Saulkalne, Latvia) and heat-treated (60 °C, 24 h) sieved (3 mm mesh size) garden soil (Biolan, Eura, Finland) 1:3 (*v*/*v*). Plants were placed in an experimental automated greenhouse (HortiMaX, Maasdijk, The Netherlands) with supplemented light from Master SON-TPIA Green Power CG T 400 W (Philips, Amsterdam, The Netherlands) and Powerstar HQI-BT 400 W/D PRO (Osram, Munich, Germany) lamps (photon flux density of photosynthetically active radiation 380 µmol m^−2^ s^−1^ at the plant level) for a 16-h photoperiod, day/night temperature of 24/16 °C, and relative air humidity of 60 to 70%. Individual containers were randomly redistributed weekly on a greenhouse bench. Substrate water content was monitored with HH2 moisture meter equipped with WET-2 sensor (Delta-T Devices, Burwell, UK) and kept at 50 to 60%. Treatment was performed after a week-long-period of additional acclimatization, with five individual plants per treatment per accession. Necessary amount of respective heavy metal salts was dissolved in deionized water and 0.1 L per container was evenly applied to soil to reach the planned final concentration of the metal (Table 2). Every third week plants were fertilized with Yara Tera Kristalon Red and Yara Tera Calcinit fertilizers (Yara International, Oslo, Norway). A stock solution was prepared for each fertilizer (100 g L^−1^) and working solution contained 25 mL of each per 10 L deionized water, used with a rate 100 mL per container.

Plants were cultivated for 9 weeks after the treatment and then the experiment was terminated. Plant shoots were carefully rinsed with deionized water to remove any soil particles or deposited salts. Plants were individually separated into different parts: leaves, flower stalks, flowers (inflorescences), and roots. Roots were thoroughly washed with water to remove any attached soil particles. Number of flowers and length of flower stalks were estimated. Both fresh and dry mass (after drying at 60 °C for 72 h) of plant parts were measured. Representative samples of all plant parts (also older leaves and younger leaves separately) were used for water content analysis (expressed as g of H_2_O per g dry mass) and analysis of heavy metal concentration.

### 2.5. Experiment 3: Soil Culture with AM3

Plants were established by seeds. Seeds were surface-sterilized with a half-diluted commercial bleach (ACE, Procter & Gamble, Warszawa, Poland) containing 5% sodium hypochlorite for 10 min, followed by three rinses with deionized water (10 min each). Prepared seeds were placed in 1 L plastic plant tissue culture containers filled with autoclaved (1 atm, 20 min) sieved (3 mm mesh size) garden soil (Biolan, Eura, Finland), closed with lids, and further cultivated for two weeks in a growth cabinet (light/dark period of 16/8 h, photosynthetically active radiation with a photon flux density of 100 µmol m^−2^ s^−1^, and day/night temperature 5/15 °C). After that, temperature regime was changed to day/night temperature of 15/20 °C and cultivated for an additional 2 weeks. Seedlings were transferred to greenhouse for another 7 weeks and then were transplanted to 0.25 L plastic containers with a mixture of quartz sand (Saulkalne S, Saulkalne, Latvia) and garden soil (Biolan, Eura, Finland) 1:3 (*v*/*v*). After 2 weeks, plants were transplanted to 0.5 L plastic containers with the same substrate and cultivated for 4 weeks. Then, plants were gradually treated with heavy metal salt solutions for 5 weeks until the planned final concentrations were reached (Table 2). Five individual plants per treatment was used.

Plants were cultivated for 9 weeks after full treatment and then the experiment was terminated. Plant shoots were carefully rinsed with deionized water to remove any soil particles or deposited salts. Plants were individually separated into different parts: leaves, flower stalks, flowers (inflorescences), and roots. Roots were thoroughly washed with water to remove any attached soil particles. Number of flowers and length of flower stalks were estimated. Both fresh and dry mass (after drying at 60 °C for 72 h) of plant parts were measured. Representative samples of all plant parts were used for water content analysis (expressed as g of H_2_O per g dry mass) and analysis of heavy metal concentration.

### 2.6. Analysis of Heavy Metals

Concentration of Cd, Cu, Mn, Pb, and Zn was measured in dried material for all plant parts. For each plant sample from soil culture experiments, approximately 1 g of dried plant tissues were collected. For analysis of metal contents from tissue culture, samples of 0.2–0.5 g were weighed. Samples were ground and plant tissue test solution was prepared by dry ashing with HNO_3_ vapor and re-dissolving in a 3% HCl solution. The testing solution was used for the determination of analyzed heavy metals. Microwave plasma atomic emission spectrometry (4200 MP-AES, Agilent, Santa Clara, CA, USA) was used for the measurement of metals according to manufacturer′s instructions. Analyzed element concentrations in plant tissue were expressed as mg kg^−1^ dry mass.

Bioconcentration efficiency was evaluated by means of bioconcentration factor, calculated as a ratio between metal concentration in growing medium and that in roots or leaves.

### 2.7. Data Analysis

For tissue culture experiment, five replicates with five explants each were used for each treatment for each accession. For soil-cultivated plants, five replicates (each with one individual plant) were used for each treatment for each accession. For heavy metal analysis, three replicate samples from randomly selected tissue culture jars or individual plants were used for each treatment for each accession.

Results were analyzed by KaleidaGraph (v. 5.0, Synergy Software, Reading, PA, USA). Statistical significance of differences for measured parameters between treatments and accessions was evaluated by one-way ANOVA using post-hoc analysis with minimum significant difference. Significant differences were indicated by *p* < 0.05.

## 3. Results

### 3.1. Experiment 1

**Figure 1:**
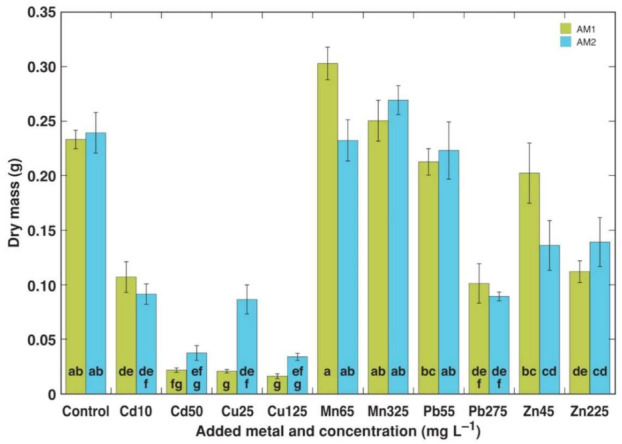
Effect of heavy metals on dry mass of plantlets of *Armeria maritima* accessions AM1 and AM2 after 4 weeks in tissue culture: Cd10, Cd 10 mg L^−1^; Cd50, Cd 50 mg L^−1^; Cu25, Cu 25 mg L^−1^; Cu125, Cu 125 mg L^−1^; Mn65, Mn 65 mg L^−1^; Mn325, Mn 325 mg L^−1^; Pb55, Pb 55 mg L^−1^; Pb275, Pb 275 mg L^−1^; Zn45, Zn 45 mg L^−1^; Zn225, Zn 225 mg L^−1^. Data are means ± SE from 5 replicates. Different letters between accessions and treatments indicate statistically significant differences (*p* < 0.05).

The change in the dry mass of the *A. maritima* plantlets due to the presence of heavy metals in the cultivation medium was used as a main criterion for their tolerance (Figure 1). Cd at 10 mg L^−1^ significantly inhibited the plantlet growth of both accessions (by 54 and 62%, for AM1 and AM2, respectively), and the effect was more severe at 50 mg L^−1^ Cd (by 91 and 84%), characterized by the absence of multiplication and an almost complete necrotization of the plantlets (Table 3). Treatment with Cu resulted in severe growth inhibition and necrotization for AM1 plantlets even at 25 mg L^−1^, but AM2 was significantly more tolerant to the lowest Cu concentration. No negative effect was seen in the Mn-treated plantlets and in the plantlets treated with 55 mg L^−1^ Pb. However, their growths were significantly inhibited at 275 mg L^−1^ Pb (by 57 and 63% for AM1 and AM2, respectively). Zn treatment significantly inhibited plantlet growth at 45 mg L^−1^ for AM2 and at 225 mg L^–1^ for both accessions (Figure 1), but the multiplication rate significantly decreased only for AM1 at the highest Zn concentration, accompanied with the appearance of some necrotic explants (Table 3). The water content in the plantlet tissues was a relatively sensitive trait with respect to heavy metal treatment, but it showed strong variation between the replicates (Table 3).

**Figure 2:**
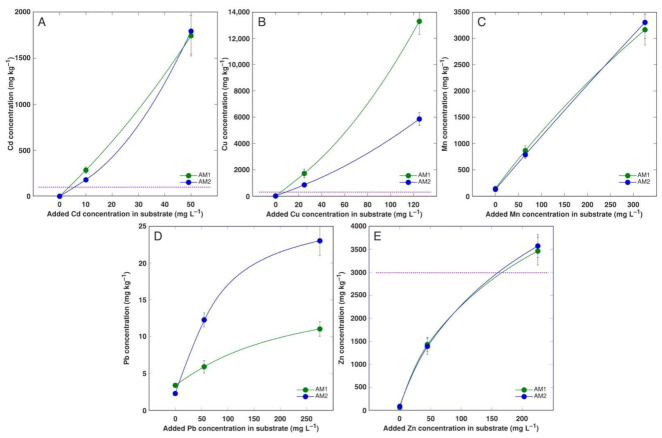
Concentration of heavy metals in plantlet tissues of *Armeria maritima* accessions AM1 and AM2 treated with heavy metals after 4 weeks in tissue culture: (**A**), cadmium; (**B**), copper; (**C**), manganese; (**D**), lead; (**E**), zinc. Data are means ± SE from 3 samples. Dotted line indicates accepted hyperaccumulation threshold level for the respective metal.

The accumulation of Cd and Mn in the plantlet tissues near linearly depended on the metal concentration in the medium and was identical for both *A. maritima* accessions (Figure 2A,C). The accumulation of Zn also was identical for both accessions, but some saturation of the response at a high medium Zn concentration was evident (Figure 2E). The Cu accumulation was extremely high and was more pronounced in the more sensitive accession AM1 (Figure 2B). The Pb accumulation response was clearly saturable, and more Pb was accumulated in the plantlets of accession AM2, but the concentration level was rather low (Figure 2D).

### 3.2. Experiment 2

When tissue culture-propagated plants were cultivated in soil, the number of decayed leaves was relatively very small and evidently was not affected by the treatments. In a quantifiable manner, this was indicated by the water content in the older leaves, and the Cu AM1 plants only showed a significant decrease in their leaf water content at 500 mg L^−1^ (Table 4). In addition, the total dry mass changed relatively little with the heavy metal treatments, with no significantly negative effects for both accessions (Table 4). However, the plant biomass significantly increased at 200 mg L^−1^ Mn for AM1 and 200 mg L^−1^ Zn for AM2. Similarly, the number of inflorescences significantly increased at 200 mg L^−1^ Mn for AM1 plants, but the total length of the flower stalks significantly increased for AM2 at 100 and 500 mg L^−1^ Cu and 200 mg L^−1^ Zn.

**Figure 3:**
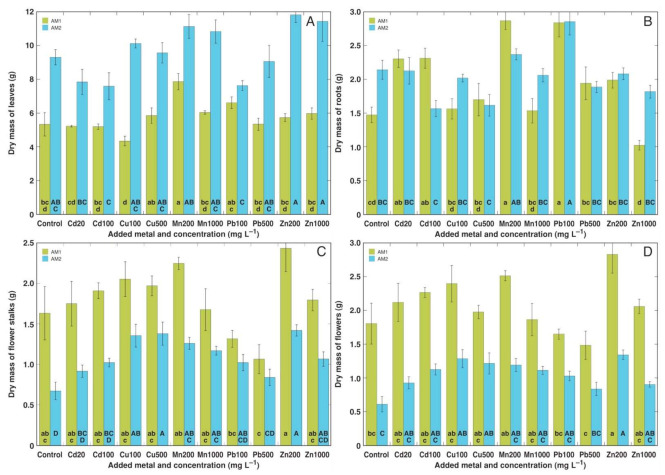
Effect of heavy metal treatment on dry mass of leaves (**A**), dry mass of roots (**B**), dry mass of flower stalks (**C**), and dry mass of flowers (**D**) of *Armeria maritima* accessions AM1 and AM2 cultivated for 9 weeks in soil. Cd20, Cd 20 mg L^−1^; Cd100, Cd 100 mg L^−1^; Cu100, Cu 100 mg L^−1^; Cu500, Cu 500 mg L^−1^; Mn200, Mn 200 mg L^−1^; Mn1000, Mn 1000 mg L^−1^; Pb100, Pb 100 mg L^−1^; Pb500, Pb 500 mg L^−1^; Zn200, Zn 200 mg L^−1^; Zn1000, Zn 1000 mg L^−1^. Different letters between treatments for a particular parameter for each accession indicate statistically significant differences (*p* < 0.05), results between AM1 and AM2 were not compared. Data are means ± SE from 5 replicates.

Differences between the two *A. maritima* accessions were especially pronounced when morphological responses to heavy metals were evaluated. In spite of the relatively similar total biomass values, there were differences in the type of biomass-partitioning activities between leaves and generative parts (Figure 3). In general, AM1 accumulated more biomass in its flower stalks and inflorescences in comparison to AM2, but AM2 accumulated more in its leaves. Due to these differences, as a result of the heavy metal treatment, only plants of the AM1 type showed a significant increase in the leaf biomass, and only plants of the AM2 type showed a significant increase in the biomass of both flower stalks and inflorescences. Thus, the dry mass of the leaves significantly increased in the AM1 plants treated with 200 mg L^−1^ Mn (Figure 3A). The dry mass of the flower stalks significantly increased in the AM2 plants treated with 100 and 500 mg L^−1^ Cu, 200 and 1000 mg L^−1^ Mn, and 200 mg L^−1^ Zn (Figure 3C). The dry mass of inflorescences significantly increased with 100 and 500 mg L^−1^ Cu, and 200 mg L^−1^ Zn (Figure 3D). In AM1, the lowest mass of both the flower stalks and flowers was evident in the Pb-treated plants, but the effect was not statistically significant due to the relatively high variability between the individuals. For the plants from the accession AM1, their root growth was significantly stimulated by 20 and 100 mg L^−1^ Cd, 200 mg L^−1^ Mn, and 100 mg L^−1^ Pb, but significantly inhibited by 1000 mg L^−1^ Zn (Figure 3B). For AM2 plants, their root growth was significantly stimulated by 100 mg L^−1^ Pb.

**Figure 4:**
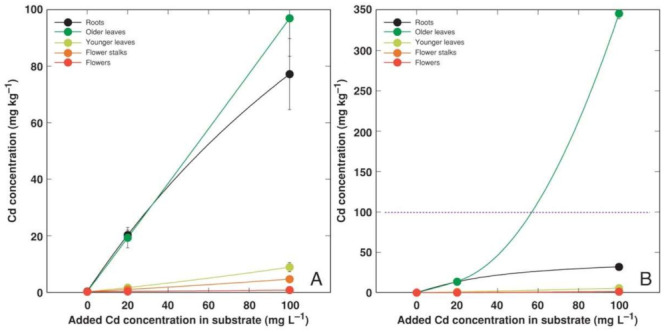
Effect of added Cd concentration in soil on Cd concentration in different parts of *Armeria maritima* plants of accession AM1 (**A**) and AM2 (**B**) after cultivation for 9 weeks. Data are means ± SE from 3 samples. Dotted line indicates accepted hyperaccumulation threshold level for the respective metal.

**Figure 5:**
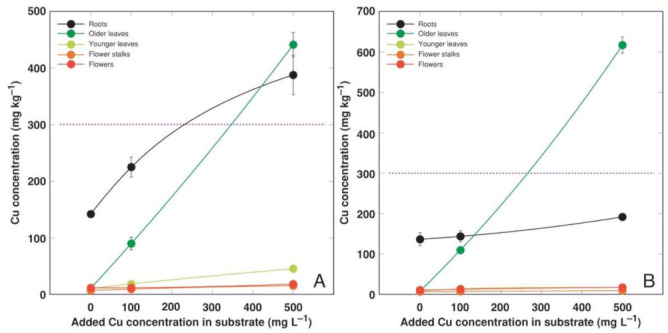
Effect of added Cu concentration in soil on Cu concentration in different parts of *Armeria maritima* plants of accession AM1 (**A**) and AM2 (**B**) after cultivation for 9 weeks. Data are means ± SE from 3 samples. Dotted line indicates accepted hyperaccumulation threshold level for the respective metal.

**Figure 6:**
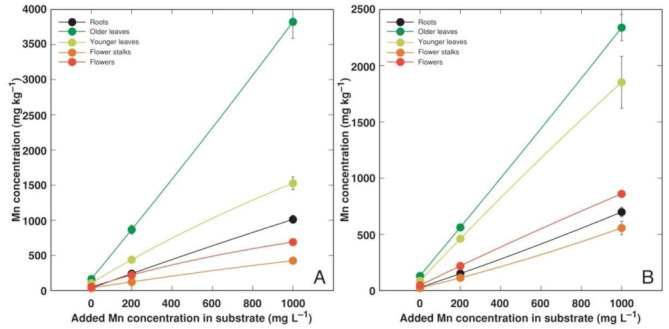
Effect of added Mn concentration in soil on Mn concentration in different parts of *Armeria maritima* plants of accession AM1 (**A**) and AM2 (**B**) after cultivation for 9 weeks. Data are means ± SE from 3 samples.

**Figure 7:**
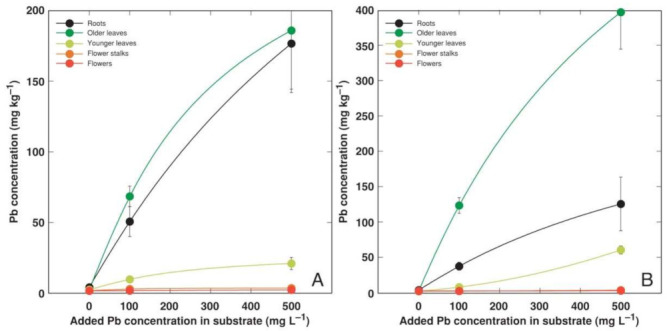
Effect of added Pb concentration in soil on Pb concentration in different parts of *Armeria maritima* plants of accession AM1 (**A**) and AM2 (**B**) after cultivation for 9 weeks. Data are means ± SE from 3 samples.

**Figure 8:**
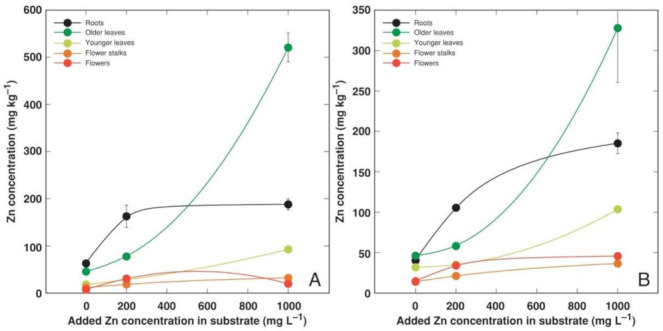
Effect of added Zn concentration in soil on Zn concentration in different parts of *Armeria maritima* plants of accession AM1 (**A**) and AM2 (**B**) after cultivation for 9 weeks. Data are means ± SE from 3 samples.

Heavy metals predominantly accumulated in the older leaves and roots of *A. maritima* plants, but different accumulation capacity and patterns were evident for particular metals as well as between the accessions. The accumulation potential for Cd was high, reaching (AM1) or even exceeding (AM2) the hyperaccumulation threshold concentration value in the older leaves, but the metal was excluded from the younger leaves or reproductive structures (Figure 4). The roots of the *A. maritima* plants showed an extreme Cu accumulation capacity even in the controlled conditions, and the hyperaccumulation threshold concentration value for Cu was exceeded in the older leaves of both accessions (Figure 5). The Mn concentration linearly increased in all the plant parts with the increasing soil Mn concentration, and the leaves accumulated more metal in comparison to the roots (Figure 6). The accumulation capacity for Pb was especially pronounced in the older leaves of AM2, but the roots of both accessions as well as the older leaves of AM1 accumulated a similar concentration of Pb (Figure 7). Pb was excluded from the generative parts. The accumulation response for Zn was saturable for the roots, and a higher Zn concentration was evident in the older leaves at 1000 mg L^−1^ Zn (Figure 8). This effect was especially pronounced for AM1.

### 3.3. Experiment 3

The growth of the *A. maritima* plants from the accession AM3 was not significantly affected by the increasing soil concentration of Cd, Mn, Pb, and Zn (Table 5). Although, the biomass of the flower stalks tended to decrease in the plants treated with high Mn and Zn concentrations. In addition, there were no significant changes in their leaf water content, indicating that the treatments did not cause increase in leaf decay rate.

**Figure 9:**
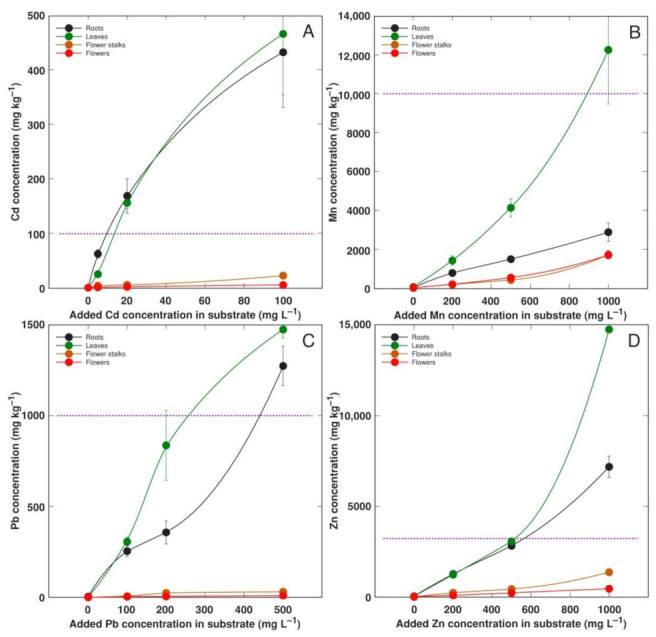
Effect of added Cd concentration in soil on Cd concentration (**A**), added Mn on Mn concentration (**B**), added Pb on Pb concentration (**C**), and added Zn on Zn concentration (**D**) in different parts of *Armeria maritima* plants of accession AM3 after cultrivation for 9 weeks. Data are means ± SE from 3 samples. Dotted line indicates accepted hyperaccumulation threshold level for the respective metal.

Plants from accession AM3 showed a high accumulation potential for Cd, Mn, Pb, and Zn in both their roots and leaves (Figure 9). The threshold concentration value for hyperaccumulation in leaves was exceeded for all metals.

### 3.4. Comparison of Bioconcentration Efficiency

**Figure 10:**
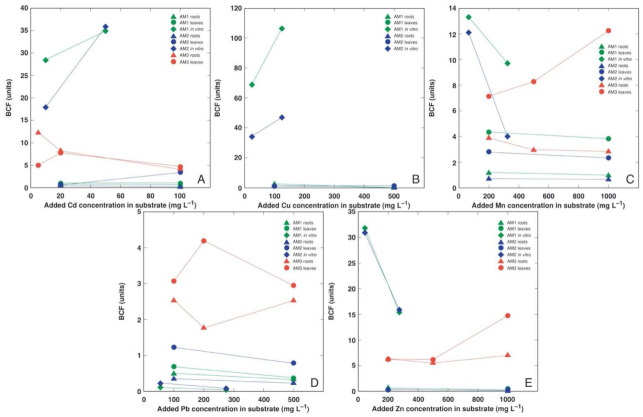
Summary of changes in bioconcentration factor for Cd (**A**), Cu (**B**), Mn (**C**), Pb (**D**), and Zn (**E**) in roots and leaves of *Armeria maritima* plants from different accessions.

The plants in the tissue culture showed a higher bioconcentration efficiency in comparison to the soil-grown plants for all heavy metals except Pb (Figure 10). Interestingly, the bioconcentration factor for Cd and Cu increased with the increasing medium metal concentration (Figure 10A,B), but it decreased for other heavy metals (Figure 10C–E). In general, the plants from the accession AM3 showed a higher bioconcentration efficiency in comparison to the AM1 and AM2 plants.

## 4. Discussion

### 4.1. Metal Tolerance of Soil-Grown Plants

In the present study with heavy metals, both essential elements for plants (Cu, Mn, Zn) as well as non-biogenous metals (Cd and Pb) were used during a relatively long-term cultivation in soil to evaluate the whole plant responses of different accessions of *A. maritima* from nonmetallicolous populations. In addition, a tissue culture experiment with roots from non-forming explants was performed for a comparison of the tissue-level tolerance and metal accumulation potential. So far, the tolerance of the different ecotypes of *A. maritima* to Cd, Pb, and Zn has been assessed in controlled conditions [15,21,29]. No studies have explored the tolerance to Mn and the accumulation potential for this metal in *A. maritima*. In addition, Cu tolerance has been assessed only with plants naturally growing in Cu-enriched soil [30,31]. In the present study, soil-grown *A. maritima* plants from different accessions showed a very high heavy metal tolerance, as the total biomass was not negatively affected by the treatments (Table 4 and Table 5).

An average range of the essential micronutrient Cu’s concentration in plant tissues is 2–20 mg kg^−1^ [32], and its concentration is tightly controlled even in metalliferous soils [33]. Toxicity symptoms usually appear above that range, with extreme differences for species with a variable Cu tolerance. Cu homeostasis is achieved by the tight control of Cu distribution and storage [34]. Surprisingly, the roots of the control plants of *A. maritima* in the present study accumulated an extremely high concentration of Cu, reaching 130 mg kg^−1^ for both AM1 and AM2, with the background of a low Cu concentration in the leaves (Figure 5). A similar concentration of Cu in the roots (141 mg kg^−1^) was noted for *A. maritima* subsp. *halleri* plants growing in highly metal-contaminated soil near a metal smelter [30]. Even roots of the Cu-hyperaccumulating species *Elsholtzia splendens* accumulated a lower concentration of Cu when cultivated in uncontaminated soil [35]. The major role of Cu in plants is established to be its participation in electron transport during photosynthesis and cellular respiration, as well as the protection it provides against oxidative stress and other processes [36], and no specific functions of Cu have been highlighted for roots. In addition to the absence of any toxicity symptoms, Cu at both concentrations even significantly stimulated the biomass accumulation in the flower stalks and inflorescences for *A. maritima* accession AM2 (Figure 3).

Mn is another essential micronutrient, with its concentration range in plant tissues usually being 1–700 mg kg^−1^ [32]. Plants’ Mn tolerance is genetically determined and shows a relatively wide range, but the lower threshold of the leaf concentration for toxicity symptoms to appear is 200 mg kg^−1^ [37]. In contrast to Cu, Mn uptake is poorly regulated [38]. Several coastal accessions of relatively salinity-tolerant species, such as *Rumex hydrolapathum, Ranunculus sceleratus,* and *Sedum maximum*, showed a high Mn tolerance and a very high accumulation potential of the metal in their leaves, reaching 6000, 7000, and 15,000 mg kg^−1^, respectively [5]. In the present study, the Mn concentration in the older leaves of *A. maritima* exceeded 12,000 mg kg^−1^ (Figure 9B). When cultivated with an excess amount of soil Mn, Mn is preferentially accumulated in older leaves due to the low mobility of Mn in phloem [39]. Among all the metals used in the present study, Mn had a relatively high bioconcentration factor for all the *A. maritima* accessions (Figure 10). It seems that in general, both the Mn tolerance and accumulation potential are high for coastal plant species from salt-affected habitats, and Mn has been argued to represent an important ecological factor in these habitats [40,41].

The high tolerance to both Cd and Zn of *A. maritima* plants of different ecotypes has already been well-established previously [15,21,29]. A prominent tolerance to these metals was also shown in the present study. Besides the absence of any negative effects on the growth of *A. maritima* plants, Cd at both concentrations significantly stimulated the root growth of the AM1 and AM2 plants (Figure 3B), but Zn at 200 mg L^−1^ significantly enhanced the biomass accumulation in the generative structures (Figure 3C,D). Non-essential toxic metals enter plant tissues through transport systems designated for the uptake of essential metal cations, as in the case of Cd, which uses Ca^2+^, Fe^2+^, and Zn^2+^ uptake systems [42]. Therefore, known hyperaccumulators of Zn can also hyperaccumulate Cd, such as *Arabidopsis halleri*, but the mechanisms of tolerance to the two metals appears to differ [43]. In a line with the former, the bioconcentration factors for Cd and Zn in the plants from *A. maritima* accession AM3 were high and relatively similar (Figure 10A,E). In the roots of *A. maritima* subsp. *halleri*, Zn accumulated in the cell walls of the rhizodermal and outer cortical cells, and this was also observed in the cell walls of leaf tissues [44]. However, it has also been shown that for the accumulation of Cd and Zn in other species, the sequestration of metals in leaf mesophyll cell vacuoles seems to be of critical importance [45].

Among the non-biogenous heavy metals, Pb represents a rather exceptional case with respect to its soil availability and the accumulation of this metal. It is argued that a relatively high Pb concentration in the leaves of plants growing in natural conditions is mostly due to environmental contamination, but an increased Pb uptake in controlled conditions can be obtained only in severely affected “unnatural” systems as hydroponics or under the effect of complexing agents in the soil [46]. Indeed, in experiments using chemical chelators, it was found that the hydroponic application of Pb in an unchelated form resulted in the metal’s accumulation in the roots of *Pinus radiata* plants, while Pb in a chelated form was transported to the above-ground parts and accumulated in the needles [47].

Most likely, Pb transport into rhizodermal cells occurs non-selectively by Ca-permeable channels and depends on an ATPase-generated proton gradient [48,49]. Further, the Casparian strip in the endodermis acts as a physical barrier, often resulting in a predominant accumulation of Pb in root tissues [48,50]. Even *A. maritima* subsp. *halleri* accumulated a significantly lower Pb concentration in its leaves in comparison to its roots [29,30,44]. However, in the present study, in soil-grown *A. maritima* plants from accession AM2, more than twice as much Pb accumulated in older leaves in comparison to roots (Figure 7B).

However, a data analysis on the effects of Pb in plants needs to be performed with special care. Lead nitrate is very often used as a source of Pb in experiments with plants without an additional control to offset the effect of surplus nitrogen. As a result, it is highly likely that any positive effect of Pb reported in this type of experiment is associated with the effect of additional nitrogen. For example, in shoot explants of *Populus alba*, lead nitrate stimulated both shoot growth and root formation [51]. Similarly, while lead nitrate resulted in a decrease in the biomass of *Dianthus carthusianorium* explants in a tissue culture, the growth tolerance index, calculated as the relative effect of the Pb on growth, increased with an increasing lead nitrate concentration [52]. Moreover, when detached leaves of *A. maritima* were immersed in a solution containing Pb(NO_3_)_2_, this resulted in an increased rate of dark respiration, which the authors only associated with the effect of Pb [53].

The immobilization by ligands and cellular sequestration are the two most likely mechanisms of heavy metal tolerance in plants [54]. In addition to the retention of heavy metals in roots, these aspects seem to also be important for heavy metal tolerance in *A. maritima* plants [19,30]. Moreover, for *A. maritima*, exhibiting the presence of salt glands on both leaf surfaces, not only Na and Cl but also other elements, including Cu, Mn, and Zn, can be actively excreted [55]. More generally, especially in the case of transition metals, an increased capacity of the enzymatic antioxidative system to counteract the formation of reactive oxygen species is of critical importance [53,56].

### 4.2. Metal Accumulation Potential of Different Accessions

The absolute higher values of the heavy metal concentrations reached in the present study in the leaves of soil-grown *A. maritima* plants belonging to a sandy-soil ecotype (500 mg Cd kg^−1^ for AM3, 600 mg Cu kg^−1^ for AM2, 12,000 mg Mn kg^−1^ for AM3, 1500 mg Pb kg^−1^ for AM3, and 15,000 mg Zn kg^−1^ for AM3) were higher than the respective threshold values for hyperaccumulation [33]. So far, the highest metal concentrations found in the leaves of *A. maritima* subsp. *halleri* plants grown in natural metal-contaminated soils were 18 mg Cd kg^−1^, 516 mg Cu kg^−1^, and 3012 mg Zn kg^−1^ [57], as well as 532 mg Pb kg^−1^ [44]. However, it needs to be emphasized that in highly contaminated habitats, a high leaf-metal concentration can result from aerial exposure [33]. In controlled conditions with substrate- or hydroponically grown plants, the respective values were strikingly lower [15,29]. The leaf Cd concentration reached 6300 mg kg^−1^ and for Pb 2107 mg kg^−1^ only when mining waste material was used as a substrate for cultivation [21].

When the soil-grown plants were compared, the *A. maritima* accession AM3 clearly showed a higher metal accumulation potential than the other two accessions. In the case of Cd, its concentration in the leaves of AM3 (Figure 9A) was similar to that for AM2 (Figure 4). However, the leaves of AM3 accumulated more Mn, Pb, and Zn than the other two accessions. While there is reason to believe that these differences were genetically-determined, an experiment with AM3 was not performed simultaneously with those involving AM1 and AM2. Therefore, some other factors might be responsible for the observed differences. Older vs younger leaves were not separately analyzed for heavy metal concentration in the experiment with *A. maritima* accession AM3. Therefore, it could be possible that the higher accumulation potential for heavy metals in the leaves of AM3 in comparison to AM1 and AM2 was at least partially associated with the higher degree of older leaves in AM3. Indeed, the average water content in all the leaves of the AM3 plants was in the range from 2.00 to 2.71 g per g dry mass (Table 5), but the respective values for the older leaves of the plants from AM1 and AM2 were 3.1–4.6 and 4.1–5.3 g g^−1^ (Table 4). It cannot be excluded, however, that the higher leaf water content in AM1 and AM2 was also related to the method used for the plant propagation, as the particular plant material was multiplicated in the tissue culture on a medium containing growth regulators. In addition, for Cd and Pb, the metal concentration in the roots was similar to that in the leaves for the accessions AM1 and AM3, but AM2 accumulated more in the leaves.

The gradient of metal accumulation in different parts of *A. maritima* has been demonstrated previously for plants from different ecological groups. In particular, when grown in a heavy metal-polluted sediment, *A. maritima* from coastal rocky cliffs preferentially accumulated Cd, Cr, Cu, and Zn in its roots, with concentrations in aerial parts being almost 10 times lower [58]. In addition, *A. maritima* ssp. *halleri* near a metal smelter showed an exclusion strategy, with the root concentrations of Pb and Cu being 20 and 88 times larger, respectively, in comparison to those in the living leaves [30]. The concentration of Zn, Cd, Pb, and Cu in the decayed leaves was three to eight times larger than that in the living leaves. In hydroponically grown plants, an identical concentration of heavy metals was accumulated in young and mature green leaves, with the concentration in decayed leaves being significantly higher, but with a several fold higher concentration in the roots [29]. However, the decayed leaves of *A. maritima* growing in a copper-rich bog accumulated more Cu than its roots [31]. In addition, other species of the genus accumulate higher concentrations of metals in their roots than in their shoots when grown in metal-contaminated soils [59,60].

In the present study, metals were clearly excluded from generative structures, but leaf and root tissues accumulated identical concentrations of heavy metals, and there were several cases where metals accumulated in higher concentrations in older leaves in comparison to that in roots: Cd for AM2, Cu for AM2, Mn for all accessions, Pb for AM2, and Zn for all accessions. It is difficult to explain the differences between the previous and the current studies, but the contamination of the leaf material can be fully excluded. Theoretically, the high metal accumulation capacity in older leaves could be associated with an increased proportion of decaying leaves; however, according to the water content analysis, this proportion was not significantly affected by heavy metal treatments for AM2 and AM3 and increased only for Cu-treated AM1 plants at the highest concentration (500 mg L^−1^) (Table 4 and Table 5). It is possible that no special attention has been paid to the age of the sampled leaves in some other studies; thus, the lower metal concentrations in the leaves in comparison to that in roots could be due to the younger age of the leaves.

### 4.3. Comparison of In Vitro and In Planta Experimental Systems

Tissue cultures have been relatively often used for the selection of heavy metal-tolerant genotypes [61,62,63]. However, there are no comparative studies available showing whether there is a sufficient consistency between in vitro and in planta metal tolerances. As we expected to observe a higher sensitivity of *A. maritima* explants in the tissue culture in comparison to the soil-grown plants, lower concentrations of the respective metals were used for the in vitro treatments. However, a higher sensitivity in the tissue culture in comparison to that in the whole plants was found only for Cd and Cu.

There is no doubt that the two experimental systems used for the accession of heavy metal tolerance and the metal accumulation potential of *A. maritima* plants differed in several of plants’ essential properties. For soil-grown plants, their roots and their surrounding microbiota act both as a barrier as well as a facilitator of the uptake of chemical substances [64]. In the present study, non-rooting explants were used as models in the tissue culture experiment, and the selected system affected the mode of acquisition of the heavy metals. The cut surface area at the lower end of the explant as well as part of the epidermis of the tissues immersed in the agar medium represent the plant–medium interface potentially involved in the nutrient uptake from the medium. There are only a few studies available so far describing the mineral nutrient uptake in in vitro cultivated plant tissues, and in general, it was noted that the concentration of minerals in plantlets was directly related to their concentration in the medium, suggesting passive diffusion as a main mechanism for mineral uptake in the absence of roots [65,66]. However, some studies indicated interactions between the uptake of different mineral elements [67], suggesting that active mechanisms might be involved that are similar to those occurring in plants ex vitro [68]. There is no doubt that immediately after explant placement in tissue culture medium, cut surfaces ensures the uninterrupted inflow of substances through apoplastic space. However, formation of wound periderm on explant cut surfaces possibly followed by development of callus-like structures [69], with suberinization of walls of outermost cell layers, can be proposed eventually suppressing apoplastic pathway and allowing for controlled influx of substances through a symplast.

The peculiarity of the current tissue culture experiment was that the *A. maritima* explants were cultivated on a multiplication medium with no root formation observed. The seemingly unrestricted uptake of Cd and Cu in the tissue culture resulted in the buildup of a high concentration of Cd (reaching 2000 mg kg^−1^; Figure 2A) and an extremely high concentration of Cu (reaching 14,000 mg kg^−1^; Figure 2B) in the explant tissues, leading to significant growth inhibition and eventually tissue necrosis (Figure 1). The most severe effects were evident for accession AM1 (Table 3), accumulating more than twice as much Cu as AM2 (Figure 2B). Interestingly, plants of the AM1 type accumulated more Cu in their leaves as in soil-grown plants (Figure 5). While the in planta accumulation potential of Cu was 12–20 times lower than that in vitro, the Cu concentration in the leaves of both accessions still exceeded the established hyperaccumulation threshold value, 300 mg kg^−1^ [30]. The accumulation potential for Mn was similar both in the tissue-cultured (Figure 2C) and soil-grown plants of AM1, but for AM2 grown in soil the leaf Mn concentration was lower (Figure 6). While the accumulation of Pb in the tissues of the cultivated explants seemed to be very restricted (Figure 2D), the leaves of the soil-grown AM2 plants still accumulated a higher concentration of Pb in comparison to AM1 (Figure 7). Similar to Cd and Cu, the Zn accumulation in in vitro conditions (Figure 2E) was also more pronounced than in the leaves of soil-grown plants (Figure 8). It is evident that plant integrity is a prerequisite for the control of the uptake of Cd, Cu, and Mn.

The accumulation of Cd and especially Cu in the plantlet tissues was rather extreme. For AM1, the higher sensitivity to Cu was clearly associated with the higher tissue accumulation capacity for this metal. At 25 mg L^−1^ Cu in the culture medium, the tissue culture of AM2 contained 850 mg Cu kg^−1^ and had 36% of the necrotic explants, but AM1 contained 1719 mg Cu kg^−1^ and already showed 100% necrotization. In contrast to the accumulation of 250 mg Cd kg^−1^ in the *A. maritima* explants at a 10 mg L^−1^ Cd concentration in the medium, the root-forming explants of the metallophytic ecotype of *Dianthum carthusianorum* accumulated only up to 33 mg Cd kg^−1^ at 5.5 μM Cd [52].

The high tolerance to Pb in the tissue culture seemed to be associated with the inability of the tissues to accumulate Pb, with Pb concentrations reaching only 25 mg kg^−1^ (Figure 2D). As the Pb concentrations in the leaves of the soil-grown *A. maritima* were significantly higher; evidently, the whole plants in soil acquire the capacity for Pb uptake due to specific root functions. Indeed, the uptake of Pb in excised leaves of *A. maritima* plants from metallicolous and nonmetallicolous populations was similar, with tissue concentration reaching 18–19 mg g^−1^ at 20 mM Pb [53]. However, Pb completely inhibited root formation in *Dianthum carthusianorum* explants with an increasing medium Pb concentration, but the accumulation of Pb continued to increase in explant tissues up to 54 mg kg^−1^ [52]. Therefore, it is also possible that other factors were responsible for the lack of Pb accumulation in the explant tissues of *A. maritima.* The easy complex formation between Pb and both inorganic anions and organic ligands is a well-known phenomenon, in general leading to the immobilization of Pb in soils and the low availability of the element for plants [48,70]. It is possible that Pb interacted with the components of the agarized medium used for the cultivation of the *A. maritima* explants. Both phosphates and carbonates are possible targets for Pb precipitation [70], but the occurrence of other possible chemical interactions cannot be excluded, especially during the autoclaving of the media [71,72]. Still, plants of AM2 type accumulated about twice as high of a concentration of Pb in their leaf tissues in comparison to AM1 plants, both in the tissue culture and soil-grown plants, pointing to the existence of inherent tissue-level differences in Pb acquisition. However, in other experiments in tissue culture, relatively high concentrations of Pb were found, as in the shoot tissues of the bromeliad species *Aechmea blanchetiana*, where an accumulation of Pb up to 600 mg kg^−1^ was evident [73]. When the root-forming explants were used for assessing the metal tolerance and accumulation potential, both Cd and Pb showed predominant accumulation levels in the explant roots. Thus, a very high concentration of Cd (up to 826 and 1514 mg kg^−1^) accumulated in the plantlet roots of *Populus tremula* × *Populus tremuloides* and *Sorbus aucuparia*, respectively, but their levels in the shoots were relatively minor (up to 126 and 3 mg kg^−1^, respectively) [23]. Pb also predominantly accumulated in the roots, reaching 13,051 and 5728 mg kg^−1^ for *Populus tremula* × *Populus tremuloides* and *Sorbus aucuparia*, respectively. No growth inhibition or visual signs of toxicity of the metals were evident, even at 56 mg L^−1^ Cd and 103 mg L^−1^ Pb [23].

### 4.4. Perspectives of High Metal Tolerance and Accumulation Potential in A. maritima

So far, *A. maritima* has only once been reported as a “hyperaccumulating species” in a one-hundred-year-old study reporting an accumulation of 1600 mg Pb kg^−1^ [74]. However, tissue concentrations of heavy metals well above the hyperaccumulation threshold concentration for the respective metals have been achieved in experiments with *A. maritima* in controlled conditions. Proponents of “true” hyperaccumulation have a very strong line against the use of various “artificial” cultivation systems, such as hydroponics with unrealistically high metal doses or standard soil spiked with soluble metal salts [33]. However, the exclusion of such approaches or even relatively more realistic experimental approaches limits the scientific interest in the phenomenon of hyperaccumulation and makes it only an ecological curiosity. The wider scientific interest in the phenomenon has been associated mostly with practical developments aimed at the recultivation of degraded environments, but there is also an immense importance of these studies with respect to the development of physiological adaptation theory or the functional aspects of ecosystem conservation, even though these applications have yet to be defined. Therefore, without denying that any soil system is more realistic for experimental plant cultivation than hydroponics, the setting that “natural populations must be studied” [33] should be treated with a healthy scientific caution, always discussing the limitations of the model system used to obtain the data and the degree of generalization associated with it.

## 5. Conclusions

The results of the current study clearly suggest that *A. maritima* represents a case of a species-wide heavy metal tolerance with a high metal accumulation potential, but substantial variation in these parameters can be found even between populations from nonmetalliferous soils. The obtained data support the hypothesis that heavy metal-tolerant populations of *A. maritima* evolved from ancestors with a relatively high basal level of metal tolerance [75]. Consequently, geographically-isolated populations of *A. maritima* are interesting for further studies both as models for understanding the physiological mechanisms of heavy metal tolerance and accumulation and as resources for practical developments aimed at their use in phytoremediation. The comparison between the results obtained in the tissue culture with root-non-forming explants with those of the soil-grown plants indicates that physiological integrity is an important feature in plants’ metal tolerance and accumulation. Tissue culture experiments seem to be useful for relative comparisons of metal tolerance between genotypes, but the results from such studies need to be interpreted with caution, as the absolute levels of tolerance for particular metals can significantly differ from soil-grown plants. In addition, the levels of heavy metals accumulating in the conditions of tissue culture, especially in the case of non-rooting explants, do not reflect metal homeostasis occurring at the whole plant level in the soil.

## Figures and Tables

**Table 1:** Accessions of *Armeria maritima* used in the present study and performed experiments.

Code	Associated Water Reservoir	Habitat	Location	Coordinates	Type of Experiment	Heavy Metals Tested
AM1	River Vecdaugava	Dry shore meadow	City of Riga, Ziemeļu District, Vecdaugava	57°03′29″ N 24°05′47′′ E	Tissue culture, soil culture	Cd, Cu, Mn, Pb, Zn
AM2	River Buļļupe	Salt-affected shore meadow	City of Riga, Kurzeme District, Island of Buļļu Sala, Vakarbuļļi	56°59′54″ N 23°57′31″ E	Tissue culture, soil culture	Cd, Cu, Mn, Pb, Zn
AM3	The Baltic Sea	Dry coastal meadow	Nybrostrand, Ystad Municipality, Skåne County, Sweden	55°25′40″ N 13°57′27″ E	Soil culture	Cd, Mn, Pb, Zn

**Table 2:** Heavy metal salts and metal concentrations in culture medium or soil used in the present study.

Heavy Metal	Salt	Added Metal Concentration (mg L^−1^)		
		Tissue Culture (Experiment 1)	Soil Culture (Experiment 2)	Soil Culture (Experiment 3)
**Cd**	CdCl_2_ 2.5H_2_O	10, 50	20, 100	5, 20, 100
**Cu**	CuSO_4_ 5H_2_O	25, 125	100, 500	–
**Mn**	MnSO_4_ H_2_O	65, 325	200, 1000	200, 500, 1000
**Pb**	Pb(CH_3_COO)_2_ 3H_2_O	55, 275	100, 500	100, 200, 500
**Zn**	ZnSO_4_ H_2_O	45, 225	200, 1000	200, 500, 1000

**Table 3:** Effect of heavy metals on plantlet water content, multiplication intensity, and percentage of necrotic explants of *Armeria maritima* accessions AM1 and AM2 after 4 weeks in tissue culture.

Heavy Metal	Concentration (mg L^−1^)	Water Content (g g^−1^ DM)		Multiplication Rate (Relative Units)		Necrotic Explants (%)	
		AM1	AM2	AM1	AM2	AM1	AM2
Control	0	8.0 ± 0.3 abcd	8.0 ± 0.3 abcd	2.88 ± 0.08 ab	2.76 ± 0.08 abc	0	0
Cd	10	4.5 ± 0.6 h	5.9 ± 0.3 efgh	0.92 ± 0.20 e	0.52 ± 0.14 ef	0	0
	50	5.6 ± 0.1 fgh	6.3 ± 0.4 cdefgh	0 f	0.08 ± 0.05 f	96	96
Cu	25	5.0 ± 0.2 gh	5.8 ± 0.3 efgh	0.40 ± 0.06 ef	1.60 ± 0.30 de	100	36
	125	5.2 ± 0.4 gh	5.2 ± 0.2 fgh	0 f	0 f	100	92
Mn	65	8.7 ± 0.4 ab	8.2 ± 0.2 abc	3.00 ± 0.00 a	3.00 ± 0.00 a	0	0
	325	9.5 ± 0.5 a	9.4 ± 0.4 a	3.00 ± 0.00 a	3.00 ± 0.00 a	0	0
Pb	55	7.7 ± 0.2 abcde	7.1 ± 0.4 bcdef	2.84 ± 0.10 ab	2.92 ± 0.05 a	0	0
	275	6.2 ± 0.3 defgh	6.2 ± 0.2 defgh	2.16 ± 0.20 bcd	2.04 ± 0.23 cd	0	0
Zn	45	8.5 ± 0.7 ab	7.6 ± 0.6 abcde	2.68 ± 0.17 abc	2.56 ± 0.29 abc	0	0
	225	6.1 ± 0.3 defgh	6.4 ± 0.2 cdefg	1.72 ± 0.05 d	2.56 ± 0.20 abc	8	0

Data are means ± SE from 5 replicates. Different letters between accessions and treatments indicate statistically significant differences (*p* < 0.05).

**Table 4:** Effect of heavy metal treatment on morphological parameters of *Armeria maritima* accessions AM1 and AM2 cultivated for 9 weeks in soil.

Heavy Metal	Concentration (mg L^−1^)	Total Dry Mass (g)		Inflorescences (*n*)		Total Length of Flower Stalks (cm)		Water Content in Older Leaves (g g^−1^ DM)	
		AM1	AM2	AM1	AM2	AM1	AM2	AM1	AM2
Control	0	10.3 ± 1.3 b	12.7 ± 0.3 bcd	13.5 ± 1.8 bc	7.7 ± 0.8 a	3275 ± 551 abc	1670 ± 232 c	4.4 ± 0.1 ab	4.5 ± 0.1 abc
Cd	20	11.4 ± 0.4 b	11.8 ± 1.0 cd	16.7 ± 1.9 abc	10.7 ± 1.0 a	4302 ± 966 ab	2383 ± 190 abc	4.2 ± 0.1 ab	4.5 ± 0.1 abc
	100	10.6 ± 0.2 ab	11.3 ± 1.0 d	14.7 ± 0.8 abc	10.8 ± 0.8 a	3504 ± 178 abc	2640 ± 146 abc	4.6 ± 0.3 a	4.1 ± 0.2 c
Cu	100	10.4 ± 0.7 b	14.8 ± 0.1 abcd	17.0 ± 1.5 abc	12.5 ± 1.1 a	3720 ± 274 abc	3325 ± 382 ab	4.3 ± 0.2 ab	4.8 ± 0.2 abc
	500	10.5 ± 0.5 ab	13.8 ± 1.0 abcd	14.0 ± 1.1 abc	11.8 ± 2.0 a	3604 ± 188 abc	3096 ± 410 ab	3.1 ± 0.4 c	4.7 ± 0.1 abc
Mn	200	15.5 ± 0.5 a	16.0 ± 0.6 ab	20.0 ± 1.0 a	11.7 ± 1.3 a	4646 ± 202 a	3031 ± 203 ab	4.4 ± 0.1 ab	5.1 ± 0.3 ab
	1000	11.1 ± 0.7 b	15.2 ± 0.8 abc	15.0 ± 1.1 abc	10.3 ± 0.9 a	3195 ± 445 abc	2697 ± 91 abc	3.9 ± 0.2 abc	5.2 ± 0.1 a
Pb	100	12.4 ± 0.4 ab	12.5 ± 0.2 bcd	12.7 ± 1.0 c	8.3 ± 0.7 a	2549 ± 210 bc	2218 ± 128 bc	3.9 ± 0.0 abc	4.7 ± 0.1 abc
	500	9.8 ± 0.7 b	12.7 ± 0.9 bcd	11.5 ± 0.7 c	10.0 ± 1.6 a	2011 ± 268 c	2191 ± 287 bc	4.0 ± 0.1 abc	4.5 ± 0.1 bc
Zn	200	13.0 ± 0.9 ab	16.7 ± 0.5 a	19.0 ± 1.8 ab	12.7 ± 0.8 a	4516 ± 487 ab	3382 ± 166 a	4.4 ± 0.1 ab	5.0 ± 0.1 abc
	1000	10.9 ± 0.5 b	15.2 ± 1.4 abc	17.8 ± 1.3 abc	9.7 ± 1.0 a	3722 ± 236 abc	2368 ± 218 abc	3.7 ± 0.1 bc	4.5 ± 0.2 bc

Data are means ± SE from 5 replicates. Different letters between treatments for a particular parameter for each accession separately indicate statistically significant differences (*p* < 0.05).

**Table 5:** Morphological paramaters of *Armeria maritima* plants (accession AM3) cultivated for 9 weeks in soil with different concentrations of heavy metals.

Heavy Metal	Concentration (mg L^−1^)	Inflorescences (*n*)	Flower Stalk Length (mm)	Leaf Dry Mass (g)	Flower Stalk Dry Mass (g)	Root Dry Mass (g)	Leaf Water Content (g g^−1^ DM)
Control	0	4.8 ± 1.0	303 ± 17	1.69 ± 0.20	1.16 ± 0.14	0.87 ± 0.15	2.54 ± 0.07
Cd	20	5.2 ± 1.2	313 ± 11	1.53 ± 0.24	1.13 ± 0.16	0.80 ± 0.09	2.40 ± 0.20
	100	4.8 ± 0.3	262 ± 34	2.06 ± 0.28	1.00 ± 0.10	0.65 ± 0.29	2.32 ± 0.19
Cu	100	6.0 ± 1.7	277 ± 7	2.50 ± 0.73	1.24 ± 0.14	0.81 ± 0.19	2.38 ± 0.11
	500	6.0 ± 0.7	279 ± 14	1.90 ± 0.23	1.14 ± 0.18	0.67 ± 0.13	2.45 ± 0.11
Mn	200	5.0 ± 0.4	282 ± 29	1.78 ± 0.28	0.90 ± 0.13	0.58 ± 0.19	2.71 ± 0.14
	1000	6.0 ± 1.1	263 ± 13	1.55 ± 0.28	0.82 ± 0.15	0.77 ± 0.07	2.42 ± 0.26
Pb	100	4.3 ± 0.5	287 ± 12	1.80 ± 0.29	0.97 ± 0.12	0.83 ± 0.14	2.27 ± 0.26
	500	4.3 ± 0.6	321 ± 18	1.62 ± 0.12	1.03 ± 0.14	1.04 ± 0.50	2.71 ± 0.14
Zn	200	5.6 ± 0.8	286 ± 6	1.64 ± 0.13	1.17 ± 0.09	0.79 ± 0.11	2.59 ± 0.06
	1000	6.3 ± 1.4	286 ± 8	1.43 ± 0.13	1.05 ± 0.15	0.47 ± 0.12	2.46 ± 0.13

Data are means ± SE from 5 replicates. There were no significant differences between the treatments (*p* > 0.05).

## Data Availability

All data reported here is available from the authors upon request.
